# Prevalence and Risk Factors of Tuberculosis Lesions in Cattle Slaughtered for Human Consumption at the Slaughterhouses in the City of Ngaoundéré, Cameroon

**DOI:** 10.1155/vmi/3520761

**Published:** 2026-07-25

**Authors:** Jean Jacques Nenba Sambo, Maurice Marcel Sandeu, Stéphane Wangba Soungou, Moise Ondua, Elie Adamou Velhima, Victor Ngu Ngwa, Bouba Adji Mohammadou, Abdoulmoumini Mamoudou

**Affiliations:** ^1^ Division of Microbiology, Agricultural Research Center, Institute of Agricultural Research for Development, P.O. Box 65, Wakwa, Cameroon, arc.sci.eg; ^2^ Department of Hygiene and Animal Foodstuffs Industry, School of Veterinary Medicine and Sciences, University of Ngaoundéré, P.O. Box 454, Ngaoundere, Cameroon, univ-ndere.cm; ^3^ Department of Microbiology and Infectious Diseases, School of Veterinary Medicine and Sciences, University of Ngaoundéré, P.O. Box 454, Ngaoundere, Cameroon, univ-ndere.cm; ^4^ Centre for Research in Infectious Diseases, LSTM-Research Unit, P.O. Box 3591, Yaoundé, Cameroon, impm-cameroun.org; ^5^ Laboratory of Molecular Biology and Biotechnology, Center for Research on Health and Priority Pathologies, Institute of Medical Research and Medicinal Plants Studies, P.O. Box 13033, Yaoundé, Cameroon, impm-cameroun.org; ^6^ Department of Food Engineering and Quality Control, University Institute of Technology, University of Ngaoundere, P.O. Box 454, Ngaoundere, Cameroon, univ-ndere.cm; ^7^ Department of Parasitology, School of Veterinary Medicine and Sciences, University of Ngaoundéré, P.O. Box 454, Ngaoundere, Cameroon, univ-ndere.cm

**Keywords:** cattle, Ngaoundere slaughterhouses, prevalence, risk factors, tuberculosis lesions, Ziehl–Neelsen staining

## Abstract

**Introduction:**

Bovine tuberculosis (bTB) is a zoonotic disease of major public health concern, particularly in developing countries such as Cameroon, where control measures are poorly implemented and there is a high risk of human infection. This study aimed to estimate the prevalence of bTB lesions in slaughtered cattle and identify the associated risk factors in slaughterhouses in the city of Ngaoundéré.

**Methodology:**

A cross‐sectional study was conducted to investigate the prevalence of *tuberculosis* lesions in two slaughterhouses between April and October 2024. Tuberculosis lesions were identified during postmortem inspections performed by veterinary inspectors on slaughtered animals, and the presence of acid‐fast bacilli (AFB) in these lesions was detected using Ziehl–Neelsen (ZN) staining. In addition, a structured questionnaire was administered to assess potential risk factors, and their associations with the prevalence of *tuberculosis* were analyzed using univariate and multivariate logistic regression models.

**Results:**

Among the 1,256 cattle examined, the overall prevalence of bTB was 5.1% (95% CI: 3.88–6.22). Suspicious lesions were detected in 64 carcasses, of which 17.14% were positive for AFB, suggestive of *Mycobacterium* spp. Regarding the multivariable analysis, only cattle origin remained significantly associated with the outcome, particularly for animals from Touboro (OR = 18.9; 95% CI: 3.3–367.1) and Mbaiboum (OR = 14.9; 95% CI: 2.5–292.2). During the study period, financial losses were estimated based on 302.5 kg of organs condemned due to tuberculosis infection, using prevailing market prices. The total losses were estimated at 541,200 CFA francs. The organs contributing to the highest financial losses were the lungs and carcasses during the study period in the surveyed abattoirs.

**Conclusion:**

This study confirmed the presence of *tuberculosis* in slaughterhouses in Ngaoundéré, with infected cattle originating from various border areas of the town. It also highlighted that the lungs and carcasses were the most frequently affected, resulting in significant financial losses for butchers. These findings raise food safety concerns in Ngaoundéré and indicate a potential risk of zoonotic transmission of the pathogen.

## 1. Introduction

Bovine tuberculosis (bTB) is one of the most significant zoonotic diseases worldwide, affecting both humans and animals. It is mainly caused by *Mycobacterium bovis*, a member of the *Mycobacterium tuberculosis* complex (MTBC). Transmission to humans occurs primarily through direct contact with infected cattle or consumption of contaminated animal products. According to the World Health Organization, approximately 2.5 million people in Africa have contracted tuberculosis, with nearly 424,000 deaths, including an estimated 147,000 new cases of zoonotic tuberculosis caused by *M. bovis* and 12,500 associated deaths [1]. It has gained increasing cross‐sectoral attention because of its ability to affect both humans and animals living in the same environment [2–6].

In Cameroon, epidemiological studies and reports of carcass condemnations at slaughterhouses indicate that the disease is present in all regions of the country, although the prevalence rates vary considerably [7]. In the Adamawa Region, bTB is among the diseases most frequently detected during postmortem inspection at abattoirs, often resulting in condemnation of carcasses or organs and substantial financial losses for actors in the beef value chain [8, 9]. Between 2007 and 2016, the regional prevalence fluctuated from 1.85% to 5.42%, with particularly high rates reported in the Vina (4.58%) and Mbéré (4.16%) Divisions [7]. Beyond its economic impact, bTB is recognized as a priority zoonosis in Cameroon [2]. Furthermore, a major challenge in public health is that infections caused by *M. tuberculosis* and *M. bovis* are clinically difficult to distinguish. While *M. tuberculosis* mainly causes pulmonary disease, both pathogens may also produce extrapulmonary forms that affect the lymph nodes and other organs. Moreover, many routine diagnostic tests used in humans do not differentiate between these two species. This limitation may contribute to the underdiagnosis of zoonotic tuberculosis and increased exposure risks among occupational groups, such as cattle farmers, slaughterhouse workers, butchers, veterinarians, healthcare personnel, wildlife workers, and immunocompromised individuals [1, 7, 10]. These challenges are compounded by insufficient surveillance data in both human and animal populations, ongoing zoonotic transmission risks, and inadequate livestock management strategies [1, 11, 12].

In cattle, diagnosis relies on several tests applied to live or slaughtered animals [13–17]. However, in most sub‐Saharan African countries, including Cameroon, detection is largely based on routine meat inspection in slaughterhouses because of limited laboratory diagnostic capacities [3, 18, 19]. Consequently, the visual examination of carcasses remains the primary method for monitoring bTB prevalence [8, 20, 21]. Data from postmortem inspections in sub‐Saharan Africa, and Cameroon in particular, reveal large fluctuations in prevalence, influenced by multiple factors, with potential socio‐economic significance for public health, as it can negatively impact national trade in animals and animal products [22–24]. The fact that the carcass is seized due to bTB, which has an impact on public health, decreases the financial capital of the farm and increases production costs. The disease also indirectly leads to losses in agricultural productivity due to the reduction in draught animals’ labor and organic manure [25].

Given the zoonotic importance of bTB, the financial losses associated with the disease, and the lack of recent epidemiological data in Ngaoundéré, updated information is required to support effective control strategies. Therefore, this study aimed to determine the prevalence of bTB based on postmortem inspection and Ziehl–Neelsen (ZN) staining of suspected tuberculosis lesions, to assess the risk factors for transmission of tuberculosis in cattle, and to estimate the financial losses due to the seizure of carcasses and organs infected with tuberculosis.

## 2. Material and Methods

### 2.1. Study Area

**FIGURE 1:**
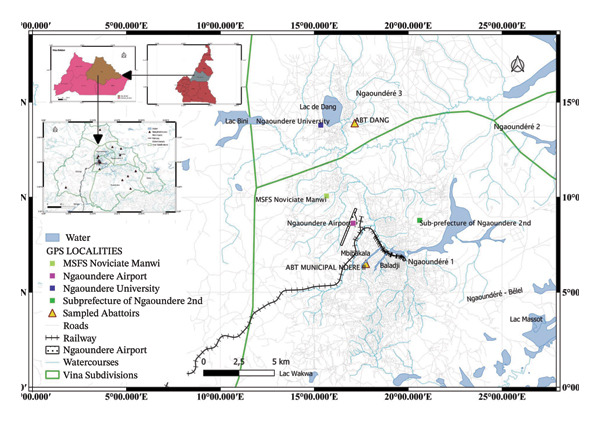
Map showing the different locations of the study area.

This descriptive cross‐sectional study was conducted from April to October in the Vina Subdivision in the Adamawa Region of Cameroon. This region is one of the main cattle production areas in Cameroon [8]. The Vina Division is located between 12.82725° and 14.76536° East longitude and between 6.52270° and 7.99075° North latitude. This division covers an area of 63,701 km^2^ and is bordered to the north by the Faro and Deo Divisions, to the south by the Mbéré Division, and to the east and west by the North Region. The study was conducted at two main slaughterhouses of the Vina Division named the municipal slaughterhouse of Ngaoundéré (MSN) and Dang slaughterhouse (DS) (Figure 1). Given that the daily number of cattle slaughtered at the selected abattoirs depended widely on the dynamics of the local livestock marketing system and transhumance movements, these abattoirs were chosen because they recorded the highest number of cattle slaughtered. This would increase our chances of detecting TB lesions.

### 2.2. Samples Collection and Identification of Tuberculosis Lesions by Postmortem Inspection

#### 2.2.1. Sample Size Determination

All slaughtered cattle were not inspected in every abattoir; both complete inspection and sampling methods were used, depending on the slaughterhouse capacity. The formula described by Thrusfield (2007) was used to determine the sample size for this study [26].

(1) N=Z2 P 1−Pd2,

where *N* = sample size, Z = appropriate value for the standard normal deviation for the desired confidence level (1.96 for 95% confidence), P = anticipated prevalence rate, Q = 1‐P, d = desired absolute precision (2%), and P = prevalence of 11.3% [27].

(2) N=1.9620.113x10.113−0.020.02427x= minimum sample.

Based on this formula, the minimum required sample size was 427. However, to improve representativeness, 1256 cattle were examined from selected abattoirs in Ngaoundéré using proportionate sampling.

Regarding animal selection, at the DS, where the daily slaughter number was low (approximately seven cattle/day), all slaughtered cattle were inspected. Then, at the MSN, where approximately 80 cattle were slaughtered daily, 20 cattle per day were selected. These animals were chosen using systematic sampling from the daily slaughter line by selecting every fourth animal (i.e., one animal after every three cattle processed) throughout the slaughter period.

#### 2.2.2. Identification of Tuberculosis Lesions by Postmortem Inspection

Slaughtering operations were conducted daily from 5:00 a.m. to 10:00 a.m. Each slaughtered animal was documented on an epidemiological data collection sheet, which recorded information such as the total number of cattle slaughtered per abattoir and the characteristics of the animals (origin, sex, and species). Anatomical and pathological parameters, including carcass appearance and lesion location, were carefully noted. Organs and tissues showing suspicious lesions were collected and examined under the supervision of veterinary inspectors to identify lesions consistent with bTB.

Tuberculosis lesions were identified using the postmortem inspection method recommended by MINEPIA and WOAH/OIE [8, 17]. This involved a thorough macroscopic anatomical and pathological examination of bovine viscera and carcasses. Routine slaughterhouse inspections included the detection of visible abnormalities, particularly tubercular lesions, in various organs, such as the lungs, liver, kidneys, uterus, spleen, udder, intestines, and associated lymph nodes. Incisions were made in the tracheal, bronchial, mediastinal, apical, medial retropharyngeal, submaxillary, mesenteric, hepatic, inguinal, and supramammary lymph nodes for detailed examination [17]. Tubercle lesions were characterized as yellowish‐white or grayish‐white granulomas encapsulated by a capsule of varying thickness, often containing caseous, caseocalcareous, or calcified material [17]. The lymph nodes were carefully sliced into thin sections using knives and examined visually under bright light to identify lesions consistent with bTB.

No animals were euthanized specifically for the purposes of this study, and no chemical euthanasia agents or dosages were used. Sample collection was performed postmortem. However, the seized carcasses were disposed of in designated pits over 20 m deep that are reserved for this purpose.

### 2.3. Prevalence of bTB Using ZN Staining

From the 64 positive lesions identified in bovine animals, 35 samples were selected using a convenience sampling approach based on the logistical availability, appearance, and location of the tuberculosis lesions to confirm the presence of acid‐fast bacilli (AFB) in cattle. These were collected in sterile containers and transported in a cooler with ice packs to the Veterinary Research Laboratory in Wakwa, which operates at biosafety level 2 (BSL‐2), for smear microscopic analysis. AFB were detected using the ZN staining technique, which highlights AFB suggestive of tuberculosis in the samples. The procedure was performed following the standard method described by the World Health Organization [28]. Without decontamination, the biopsies were manually crushed using a tissue grinder and centrifuged at 3500 rpm for 5 min. The samples were then spread and fixed on slides previously labeled according to the WHO method described by Dupeyron [29, 30]. Smear microscopy for AFB was performed using the WHO‐IUATLD grading scale [31].

### 2.4. Determination of the Financial Losses From the Seizure of Infected Organs and Carcasses

The direct financial losses (DFL) due to seizures of organs infected with bTB were assessed through anatomical identification and weight measurement (kg) of the affected organs and/or tissues, using the formula: DFL = (number of carcasses/organs condemned × weight of carcasses condemned per kg) × average price per CFA franc/kg [32]. The average market price of each seized organ was estimated through interviews with butchers and consultations with the regional chamber of commerce [33, 34]. These data were used to calculate the total financial loss resulting from the condemnation of the organs.

### 2.5. Statistical Analysis

Data were entered using Microsoft Excel software and analyzed using SPSS software (Version 20.0). The chi‐square test was used to assess significant differences in prevalence between localities, sex, and age groups of animals (*p* < 0.05). Logistic regression (univariable and multivariable) was performed to identify the associated risk factors. Prevalences were calculated by relating the number of positive cases to the total number of animals tested in each locality.

### 2.6. Ethical Considerations

The study protocol was approved by the scientific research and ethics committee of the School of Veterinary Medicine and Sciences of the University of Ngaoundéré, Cameroon (2024/145UN/ESMV/DAARCS/SSFC of April 05, 2024).

## 3. Results

### 3.1. Prevalence of Tuberculous Lesions in Slaughterhouses From bTB

Of the 1256 cattle slaughtered and inspected, 64 were infected with bTB, corresponding to an overall prevalence of 5.1% (95% CI: 3.88–6.22). By slaughterhouse, the prevalence was 5.4% (6/112) in the DS (Ngaoundéré 3rd) and 5.1% (58/1144) in the MSN. This prevalence was higher in females (3.82%), Red Fulani cattle (3.03%), and the 5–7 years age group (3.05%). Regarding organ distribution, most lesions were observed in the lungs (2.38%), followed by the lymph nodes (0.95%) and udders (0.71%) (Table 1).

**TABLE 1:** Overall prevalence of tuberculous lesions according to cattle characteristics.

Characteristics of slaughtered cattle		Number of cattle inspected (*n*)	Sample collected at postmortem examination (*n*)	Distribution of overall prevalence
Breed	Gudali	297	6	0.48%
	Red Fulani	602	38	3.03%
	White Fulani	357	20	1.59%

Age	2–4 years	22	3	0.24%
	5–7 years	948	44	3.50%
	8–10 years	286	17	1.35%

Sex	Female	884	48	3.82%
	Male	372	16	1.27%

Location of tuberculosis lesions	Lungs	1256	30	2.38%
	Liver	1256	1	0.08%
	Spleen	1256	1	0.08%
	Trachea	1256	2	0.15%
	Udder	884	9	1.01%
	Head	1256	5	0.39%
	Lymph nodes	1256	17	1.35%
	Viscera	1256	3	0.23%
	Carcass	1256	1	0.08%

*Note: n* = number.

Infected cattle mainly originated from towns within the Adamawa and North Region, as well as from localities bordering the neighboring countries. The highest prevalence was recorded in cattle from Touboro (12.6%) and Mbaiboum (10.14%) in the Mayo‐Rey Division (North Region), a key border area with Chad and the Central African Republic. Other affected localities included Meiganga (2.63%), Tike (2.33%), Dang (2.05%), Banyo (0.95%), and Telo (0.88%) in the Adamawa Region (Table 2). These sites are distributed across the Mbere, Faro‐et‐Déo, Vina, and Mayo‐Banyo Divisions.

**TABLE 2:** Distribution of prevalence according to the origin of cattle infected with bovine tuberculosis.

Origin of cattle	Number of cattle infected (*n)*	Positive number at postmortem examination (*n*)	Prevalence (95% CI)
Touboro	246	31	12.6% [8.45–16.75]
Tike	129	3	2.33% [0.27–4.93]
Mbaiboum	207	21	10.14% [6.03–14.25]
Banyo	105	1	0.95% [0.91–2.81]
Dang	195	4	2.05% [0.06–4.04]
Telo	114	1	0.88% [0.83–2.59]
CAR	38	1	2.63% [2.46–7.72]
Meiganga	38	1	2.63% [2.46–7.72]
Unknown	184	1	0.54% [0.52–1.60]
Total	1256	64	5.1% [3.88 – 6.32]

*Note: n* = number; CAR = Central African Republic.

Abbreviation: CI: confidence interval.

**FIGURE 2:**
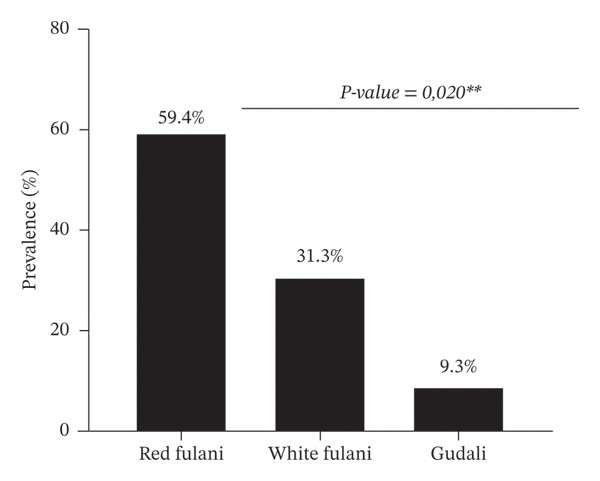
Distribution of prevalence according to the cattle breeds infected with TB.

Breed‐specific analysis showed that among the 64 positive cases, the highest prevalence was in Red Fulani (59.4%), followed by White Fulani (31.3%) and Gudali (9.3%) cattle. A statistically significant association (*p* < 0.02) was found between cattle breed and the presence of tuberculous lesions (Figure 2).

Finally, of the 64 positive animals reported, 35 samples were analyzed to confirm the presence of AFB in cattle. ZN staining revealed a prevalence of 17.14% (6/35), with these samples showing more than two AFB per 100 fields. In contrast, 82.86% (29/35) of the samples tested negative.

### 3.2. Macroscopic Lesions of bTB Detected in Different Organs of Slaughtered Cattle

**FIGURE 3:**
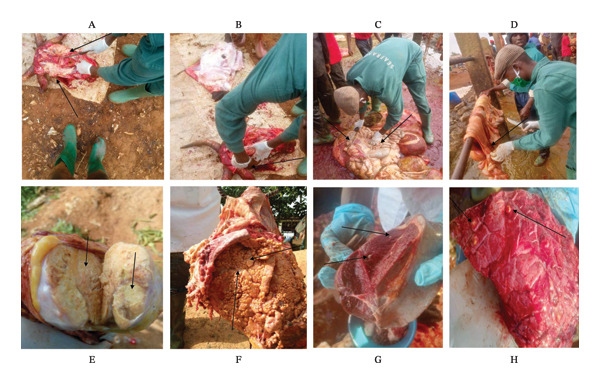
Inspection of the head for tuberculosis lesions on the submandibular (A) and parotid (B) lymph nodes; inspection of the abdominal viscera for tuberculous lesions on the gastric and hepatic lymph nodes (C) and the mesenteric lymph nodes (D); types of tuberculosis lesions on the retromammary lymph node (E), on the precural node (F), on the thoracic cavity (G), on the spleen (H).

Of the 64 slaughtered cattle examined, circumscribed and well‐delimited lesions with altered appearances and varying shapes were observed. These included caseous and miliary types (Figure 3E, F). Lesion sizes ranged from small nodules (2–3 mm), commonly distributed in the lungs (Figure 3G, H), to larger nodules measuring up to 3 cm in diameter. Additional lesions were detected in the abdominal viscera and udders (Figure 3C, D), while the affected lymph nodes were in the retropharyngeal, prescapular, mediastinal, and inguinal regions (Figure 3A, B).

### 3.3. Risk Factors Associated With the Prevalence of Tuberculous Lesions of Slaughtered Cattle

The univariable analysis showed a significant association between the prevalence of bTB detected during postmortem inspection and several risk factors related to slaughtered cattle (Table 3). The most affected breeds were Red Fulani (6.31%) and White Fulani (5.60%), compared to Gudali (2.02%). The odds of bTB were significantly higher in Red Fulani (OR = 3.2; 95% CI: 1.4–8.6) and White Fulani (OR = 2.8; 95% CI: 1.2–7.9) than in Gudali. Regarding sex, 5.43% of females were affected compared to 4.3% of males; however, the odds ratio of 0.78 (95% CI: 0.41–2.28) indicated no significant difference between sexes (*p* = 0.40). With respect to origin, the highest prevalence was recorded in cattle from Touboro (12.6%) and Mbaiboum (10.14%), both of which are located in the Mayo‐Rey Division. Based on the extremely wide 95% confidence intervals reflected in Table 3, the sample size was small. However, these results demonstrated a highly significant association between cattle origin and bTB prevalence (*p* *< 0.0001*), with the odds of infection being 15.0 (95% CI: 3.1–268.7) and 11.7 (95% CI: 2.4–211.9) times higher in Touboro and Mbaiboum, respectively, compared to Banyo (reference locality). Furthermore, the multivariate analysis confirmed that cattle originating from Touboro (*p* *< 0.00*) and Mbaiboum (*p* *< 0.01*) had increased odds of bTB infection, at 18.98 (95% CI: 3.3–367.1) and 14.9 (95% CI: 2.5–292.2), respectively, compared to Banyo (Table 4).

**TABLE 3:** Result from univariable logistic regression analysis displaying the relationship between bTB and risk factors linked to inspected cattle.

Risk factors (*n* = 1256)	Category	Positive, *n* (%)	Odds ratio (95% CI)	*p*‐value
Sex	Male	48 (5.4)	0.78 (0.42–1.37)	0,40
	Female	16 (4.3)	1,00	

Age	2–4 years	3 (13.6)	1,00	0,21
	5–7 years	44 (4.6)	0.31 (0.10–1.34)	
	8–10 years	17 (5.9)	0.40 (0.12–1.82)	

Breed	G	6 (2.0)	1,00	< 0.01^∗^
	RF	38 (6.3)	3.27 (1.47–8.67)	
	WF	20 (5.6)	2.88 (1.21–7.95)	

Abattoirs	DS	6 (5.4)	1,00	0,90
	MSN	58 (5.1)	0.94 (0.43–2.50)	

Origin	Banyo	1 (0.95)	1,00	< 0.0001^∗∗∗^
	Dang	4 (2.05)	2.17 (0.32–42.90)	
	Unknown	1 (0.54)	0.57 (0.02–14.50)	
	Mbaiboum	21 (10.14)	11.74 (2.40–211.98)	
	Meiganga	1 (2.63)	2.81 (0.11–72.30)	
	CAR	1 (2.63)	2.81 (0.11–72.30)	
	Telo	1 (0.88)	0.92 (0.04–23.50)	
	Tiké	3 (2.33)	2.50 (0.31–50.46)	
	Touboro	31 (10.60)	15.00 (3.15–268.74)	

*Note:* % = percent; CAR = Central African Republic. ^∗^: statistically significant; ^∗∗^: highly significant; ^∗∗∗^: extremely significant in this table to indicate the significance thresholds of *p* < 0.05; *p* < 0.005; and *p* < 0.005, respectively.

Abbreviations: bTB: bovine tuberculosis; CI: confidence interval; DS: Dang slaughterhouse; F: female; G: Gudali; M: male; MSN: municipal slaughterhouse of Ngaoundéré; OR: odds ratio; RF: Red Fulani; WF: White Fulani.

**TABLE 4:** Factors retained in the final multivariable mixed‐effects logistic regression model of risk of bovine tuberculosis in abattoirs of Ngaoundéré.

Risk factor	Category	Estimate	SE	Odds ratio (95% CI)	*p*‐value
Breed	G	Reference	—	1,00	—
	RF	−0,33	0,61	0.71 (0.21–2.42)	0.58
	WF	−0,51	0,63	0.60 (0.17–2.11)	0.41

Origin	Banyo	Reference	—	1,00	—
	Dang	0,84	1,13	2.32 (0.33–46.01)	0.45
	Unknown	−0,38	1,45	0.70 (0.02–18.15)	0.79
	Mbaiboum	2,70	1,10	14.98 (2.57–292.20)	< 0.01^∗^
	Meiganga	1,03	1,43	2.79 (0.11–72.01)	0.47
	CAR	1,27	1,16	3.60 (0.13–99.40)	0.40
	Telo	−0,07	1,10	0.93 (0.40–23.67)	0.95
	Tiké	0,93	0,61	2.55 (0.32–52.21)	0.42
	Touboro	2,94	0,63	18.95 (3.33–367.12)	< 0.001^∗^

*Note:* ^∗^: statistically significant; ^∗∗^: highly significant; ^∗∗∗^: extremely significant in this table to indicate the significance thresholds of *p* < 0.05; *p* < 0.005; and *p* < 0.005, respectively.

Abbreviations: CAR= Central African Republic; G = Gudali; RF= Red Fulani; SE = standard error; WF= White Fulani.

### 3.4. Estimated Financial Losses (in CFA Francs) Resulting From Carcass and Organ Seizures due to bTB During the Study Period

The financial impact of carcass and organ seizures due to bTB was evaluated to quantify the economic burden on the local beef sector. Losses were estimated based on the number of condemned carcasses and organs combined with prevailing market prices, highlighting the economic consequences for butchers and livestock owners. During the study period, the total weight of confiscated carcasses and organs infected with tuberculosis was 302.5 kg. The organs responsible for the highest financial losses were the lungs (252,000 CFA francs) and the carcasses (130,000 CFA francs) (Table 5). Overall, the total financial loss attributable to bTB was estimated to be 541,200 CFA francs during the study period for the slaughterhouses surveyed (Table 5).

**TABLE 5:** Organs seized from cattle with tuberculosis in the study and estimation of financial losses.

Condemned carcass & organs	Number of condemnations	Weight (kg)	Percentage (%)	Price (CFA franc) per 1 kg	Estimated financial losses (CFA franc)
Lungs	30	180	50.85	1400	252,000
Liver	1	7	1.69	2400	16,800
Udder	9	20	15.25	2000	40,000
Spleen	1	1.5	1.69	1400	2100
Trachea	2	1	3.40	500	500
Abdominal viscera	3	10	5.08	1400	14,000
¼ carcass	1	50	1.69	2600	130,000
Lymph nodes	12	33	20.34	2600	85,800
Total	59	302.5	100	14,300	541,200
***1 U.S. dollar = 561.28 CFA franc***					

*Note:* % = percentage; kg = kilogram; CFA franc = Central African CFA franc (XAF). Values in bold correspond to the overall parameters examined in this table.

## 4. Discussion

Tuberculosis is a major zoonotic disease with worldwide distribution [35]. bTB is a chronic infectious disease that primarily affects cattle but can also infect other domestic and wild animals and humans. Therefore, veterinary inspection of slaughterhouses is crucial to minimize the risk of transmission to humans through the consumption of infected meat. In Cameroon, the government has implemented measures to control the spread of bTB, including regular screening, slaughter of infected animals, and milk pasteurization to reduce the risk of human infection. However, these efforts face challenges, such as limited resources, lack of awareness among farmers and veterinarians, and difficulties in implementing effective surveillance and control measures in remote areas [36]. Meat inspection data represent a valuable source of information for epidemiological surveys and play an important role in the field of preventive veterinary medicine. Despite this, only a limited number of studies have investigated the monitoring of tuberculosis in slaughterhouses in Ngaoundéré. The present study aimed to determine the prevalence of bTB based on postmortem health examinations and to highlight the presence of AFB in tuberculous lesions.

The overall prevalence recorded during postmortem inspection was 5.1%. Former studies reported prevalences of up to 4.25% in three abattoirs in Cameroon [21]. In an African context, the prevalence of lesions suggestive of bTB was 1.75% and 2.7% in Burkina Faso [25, 37] and 5% in Algeria [38]. However, they were 25.73% in Burkina Faso [39] and 17% in Ethiopia based on the intradermal skin test in the herds [40]. This variation of the prevalence rate could be attributed to several factors, notably differences in diagnostic method, study design, livestock management systems, and sample size. Other contributing factors include the competence and monitoring of veterinary inspectors, requiring a considerable workload and proven expertise to avoid false negatives; and the evolution of bTB because not all infected cattle can develop macroscopic lesions during slaughtering operations, and immune conservation does not necessarily indicate clinically apparent infection [37, 41]. Also, the absence of a “test and slaughter” policy and compensation for cattle owners in many African countries, such as Cameroon, allows the disease to persist in an endemic manner [42, 43]. Regarding the distribution of prevalence, the study found that females (3.82%) in the 5–7 years age group (3.5%) had the highest infection rates. The results revealed no significant association between age and sex groups related to the prevalence rate. These findings differ from those reported in Zamfara State, Nigeria, where out of 226 carcasses observed with TB lesions, 147/226 were more infected than males. Similarly, based on the age, 149/226, the most infected were old [44]. This difference could be explained on the one hand by sample size and on the other hand by the fact that older cows are more frequently slaughtered due to their reduced reproductive and milk production potential [38]. Also, the insidious and asymptomatic nature of bTB, which progresses with age and gender, often appears to the naked eye in older cows, making them more vulnerable due to a weakened immune system [45, 46]. Moreover, the longer an animal lives, the higher its chances of exposure and infection [38].

With respect to the localization of tuberculous lesions, most were observed in the lungs and thoracic lymph nodes (2.38%). This is consistent with the findings of Konate et al. (2024), who also reported the highest proportions in the lungs and associated lymph nodes [16]. This result suggests that the lungs, through inhalation of bacteria via the respiratory tract, constitute the primary route of bTB transmission in cattle and serve as a major site for the excretion of infectious tubercle bacilli [47]. After the lungs and udders, the abdominal viscera were the most significantly affected organs. This finding differs from that of Oumar et al. (2022), who reported the liver, kidneys, and head as the most affected. Such variability in the distribution of tuberculous lesions may be explained by the ability of *M. bovis* to disseminate through the blood and lymphatic systems, thereby reaching multiple tissues and organs [38, 48].

Table 3 revealed that infections were more prevalent among the Red Fulani (38/64) and White Fulani (20/64) breeds compared to the Gudali breed (6/64). The Red Fulani (OR = 2.8; 95% CI: 1.2–7.9) and White Fulani (OR = 3.2; 95% CI: 1.4–8.6) breeds were more likely to be positive for bTB compared to the reference breed. This finding is consistent with a study conducted in Cameroon, which reported a higher prevalence of bTB in local breeds accustomed to extensive husbandry systems compared to exotic breeds and their crossbreeds [49]. Similarly, in Bafoussam, prevalence was 9% for the White Fulani breed and 4.8% for the Red Fulani breed [22]. The Zebu Fulani breeds are characteristic of the semiarid Sahelian Regions, but trade and transhumance in search of better pastures and water resources have facilitated their presence in other areas [30, 61, 59]. Regarding the origin from Table 3, we recorded the highest proportions for locations of Touboro (OR = 15; 95% CI: 3.15–268.74) and Mbaiboum (OR = 11.74; 95% CI: 2.40–211.98), compared to other locations in the Adamawa Region, with a higher chance of having infected cattle. This observation aligns with previous studies linking high bTB prevalence to areas with active commercial livestock exchanges, transhumant herds, and regions where animals share abundant pastures and water resources [36, 50–52].

Although the breed showed no significant association in Table 4, reflecting a likely small sample size, results showed that the White Fulani breed had a slightly higher chance of showing the most TB lesions compared to the Red Fulani breed. This observation differs from the former findings by Awah‐Ndukum et al. (2010) in three abattoirs of Cameroon. It also disagrees with the result obtained by Ahmad et al. (2017) in Nigeria, where the lower prevalence rate was recorded in the Red Fulani (8.4%) compared to the Sokoto Gudali (34.5%) and White Fulani (57.1%). The predominance of infections in Fulani Zebu breeds may firstly reflect the availability of this local breed to the detriment of others. Secondly, the extensive cattle production system in the Sahel is characterized by mostly the Zebu Fulani breed, which is adapted more to the long seasonal migratory movement than other local breeds in search of optimal pastures and water sources in the northern region of South Saharan Africa [22, 25, 53, 54].

Regarding the origin of the slaughtered animals, Table 4 reported significant associations for the localities of Mbaiboum (*p* *< *0.01) and Touboro (*p* *< *0.00), although, despite 95% confidence intervals, the odds ratios are extremely wide, reflecting a small sample size. These results would likely be due to the high level of animal mobility in these localities, which is an essential practice for life in pastoral environments. The movement of animals is primarily driven by the need to access natural resources and meet commercial demands. However, this mobility often occurring outside formal regulatory channels can lead to both sanitary and nonsanitary challenges. Notably, it contributes to the spread of transboundary diseases such as bTB. Veterinary services at border points, which are responsible for ensuring animal health and food safety, often operate under significant constraints. These include limited human, financial, and material resources, compounded by inadequate regulatory frameworks. As a result, key functions such as integrated surveillance systems, diagnosis, and reporting of zoonotic diseases, as well as vaccination coverage of at‐risk populations (veterinarians, abattoir workers, eco‐guards, farmers, shepherds, etc.), the border control of animals and animal products, and the enforcement of quarantine procedures are negatively affected [6, 12, 55, 56].

Although the effects of sex (*p* > 0.05; *p*‐value = 0.406) and age (*p* > 0.05; *p*‐value = 0.126) were not statistically significant, prevalence was slightly higher in females (5.43%) than in males (4.30%). The 5–7 years age group had three times the odds of being positive at postmortem inspection compared to the 2–4 years reference group, while the 8–10 years age group had twice the odds. The results revealed no significant association between age and sex groups related to the prevalence rate. These findings differ from those reported in Zamfara State, Nigeria, where out of 226 carcasses observed with TB lesions, 147/226 were more infected than males. Similarly, based on the age, 149/226, the most infected were old [44]. This could be explained by the fact that the slaughtered females were most often old and had calved many times and, due to the stress induced by successive pregnancies, would decrease the immune defense of females more than males, thus making them more vulnerable to present tuberculous lesions [38, 44].

After confirmation of suspected lesions using ZN staining, the prevalence of AFB was 17.14% (6/35). This is similar to previous studies in Cameroon, where ZN‐based prevalence was 19.11% (13/68) in 2010 [21]. However, this result differs from that of Agbalaya et al. (2020), conducted in Nigeria, which was 7% [57]. This variation in prevalence rates could be explained by several factors, notably the sensitivity of the ZN staining technique, requiring high bacillary loads (10^4^–10^5^ AFB/mL) to be positive; the quality of the sample, depending on the location of the TB lesion taken; the reader’s experience; and misclassification of lesions during gross examination. These observations align with those of Awah‐Ndukum et al. (2010; 2012), who reported that the mediastinal and bronchial lymph nodes were often more loaded with bacilli than other organs, as well as the rigor during gross examination. Also, those of Berg, 2009, focusing on the reader’s experience and the reading time of the slides [58].

During the study period, the total quantity of losses due to the seizure of carcasses and organs infected with tuberculosis was 302.5 kg, corresponding to an estimated DFL of 541,200 CFA francs. This result is closer to that reported by Oumar et al. (2022), estimated at 206,949.968 CFA francs for a study period of 4 months. However, it differs more from the one reported in Yaoundé, Cameroon, by Awah‐Ndukum et al. (2012), estimating the DFL at 6,189,650 CFA francs during a 3‐month study period. This discrepancy may be attributed to several factors, including the study design, the number of slaughterhouses interviewed, differences in postmortem sanitary inspections, the types and number of seizures, variation in prices per kilogram, and the duration of the respective studies [25, 34].

### 4.1. Study Limitation

While providing significant insights into the prevalence of bTB in Ngaoundéré, this study is subject to several methodological and analytical limitations that warrant consideration:

1. Reliance on macroscopic postmortem inspection: The primary screening for TB lesions relied on routine meat inspection. Although this is a standard practice in resource‐limited settings, it is well documented that macroscopic examination lacks optimal sensitivity. The absence of visible lesions does not definitively rule out infection, particularly in early‐stage or latent cases, which may lead to an underestimation of the true prevalence.
2. Suboptimal confirmed sampling for microscopy: Due to logistical and technical constraints, ZN staining was performed on only 35 of the 64 carcasses identified with suspicious lesions (approximately 55%). This partial sampling limits the robustness of the microscopic confirmation and may affect the overall correlation between gross pathological findings and laboratory‐confirmed AFB presence.
3. Absence standard confirmatory tests: A major limitation of this study is the lack of mycobacterial culture and molecular diagnostic tools, such as PCR or spoligotyping. Consequently, the definitive identification of *M. bovis* could not be established. While ZN staining detects AFB, it cannot differentiate between members of the MTBC and nontuberculous mycobacteria (NTM). Furthermore, the low sensitivity of ZN staining (requiring 104–105 bacilli/mL) likely contributed to the low confirmation rate (17.14%) observed among the sampled lesions.
4. Statistical and analytical constraints: Certain risk factors, particularly specific animal origins, exhibited extremely wide 95% confidence intervals for the calculated odds ratios. This suggests a limited sample size within these specific subcategories, which reduces the precision of the point estimates and necessitates a cautious interpretation of these associations.

## 5. Conclusion

The findings of the present study provide baseline data on cattle tuberculosis in Ngaoundéré, Cameroon. The prevalence was 5.1% based on postmortem inspection and 17.14% based on ZN staining, confirming the presence of AFB suggestive of the presence of *Mycobacterium* spp. in cattle slaughtered at the MSN and DS. Infection occurrence was influenced by animal‐related risk factors. The study also highlights substantial financial losses resulting from the seizure of infected carcasses and organs. Therefore, there is an urgent need for enhanced surveillance that incorporates environmental factors to assess tuberculosis exposure risks among slaughterhouse workers. Furthermore, to develop effective control programs, additional investigations are needed, including (i) molecular analysis of *Mycobacterium* strains from animals; (ii) assessment of their genetic diversity; and (iii) identification of molecular markers of drug resistance.

## Author Contributions

Stéphane Wangba Soungou, Jean Jacques Nenba Sambo, and Maurice Marcel Sandeu conceived and designed the study; Stéphane Wangba Soungou and Jean Jacques Nenba Sambo collected the data in the field; Stéphane Wangba Soungou and Jean Jacques Nenba Sambo performed the data analysis; Jean Jacques Nenba Sambo and Maurice Marcel Sandeu wrote the manuscript with contribution from Moise Ondua, Victor Ngu Ngwa, Bouba Adji Mohammadou, and Abdoulmoumini Mamoudou.

## Funding

This work was funded by the Department of Microbiology and Infectious Diseases of the School of Veterinary Science and Medicine.

## Conflicts of Interest

The authors declare no conflicts of interest.

## Data Availability

Data will be available on reasonable request.
